# Monolayer Graphene Terahertz Detector Integrated with Artificial Microstructure

**DOI:** 10.3390/s23063203

**Published:** 2023-03-17

**Authors:** Mengjie Jiang, Kaixuan Zhang, Xuyang Lv, Lin Wang, Libo Zhang, Li Han, Huaizhong Xing

**Affiliations:** 1 State Key Laboratory for Modification of Chemical Fibers and Polymer Materials, Department of Optoelectronic Science and Engineering, Donghua University, Shanghai 201620, China; 2 State Key Laboratory for Infrared, Physics Shanghai Institute of Technical Physics, Chinese Academy of Sciences, 500 Yu-tian Road, Shanghai 200083, China; 3 Hangzhou Institute for Advanced Study, College of Physics and Optoelectronic Engineering, University of Chinese Academy of Sciences, No. 1, Sub-Lane Xiangshan, Xihu District, Hangzhou 310024, China

**Keywords:** microstructure, monolayer graphene, terahertz detector

## Abstract

Graphene, known for its high carrier mobility and broad spectral response range, has proven to be a promising material in photodetection applications. However, its high dark current has limited its application as a high-sensitivity photodetector at room temperature, particularly for the detection of low-energy photons. Our research proposes a new approach for overcoming this challenge by designing lattice antennas with an asymmetric structure for use in combination with high-quality monolayers of graphene. This configuration is capable of sensitive detection of low-energy photons. The results show that the graphene terahertz detector-based microstructure antenna has a responsivity of 29 V·W^−1^ at 0.12 THz, a fast response time of 7 μs, and a noise equivalent power of less than 8.5 pW/Hz^1/2^. These results provide a new strategy for the development of graphene array-based room-temperature terahertz photodetectors.

## 1. Introduction

Terahertz (THz) waves are characterized by frequencies ranging from 0.1 to 10 THz, spanning the region between infrared and microwave regions of the electromagnetic spectrum. The distinct optical and electrical properties of these waves make THz technology highly promising for a range of applications in various domains, such as biomedicine, information security, environmental monitoring, security inspection, non-destructive testing, and defense technology [1,2,3,4,5,6]. Despite this potential, the development of THz technology has been hindered by limitations in both THz sources and highly sensitive detectors. Recent advancements in material growth and micro-/nano-fabrication techniques have led to rapid development in these areas and increasing interest from researchers. As a critical component in the THz field, the challenge of efficient photodetection and fast response remains [7]. Currently, several commercial detectors are available, including GaN detectors, GaAs-based detectors, and thermal detectors, yet their response times being in the millisecond range limits their application in the field of THz technology [8,9,10,11,12,13].

Graphene, a seminal two-dimensional material composed of monolayer carbon atoms, has garnered significant interest due to its distinctive structural attributes. Boasting high strength, exceptional conductivity, elevated carrier mobility (2 × 10^5^ cm^2^/V·s) for monolayer graphene, a bandgap of zero, broad spectral response, and limited surface dangling bonds, it has garnered significant attention in the field of photonics [14,15,16,17,18,19]. The exceptional properties of graphene make it well-suited for use in visible light, infrared, and THz frequency regimes, offering high-sensitivity detection capabilities [20,21,22]. Additionally, the small electron heat capacity of graphene leads to a substantial temperature change and elevated photocurrent upon absorbing the same amount of heat. The extension of graphene to the THz band affords its use as a material of choice in THz photodetectors, as it facilitates the photothermal effect. As a result, graphene stands out as a highly promising material for the development of THz photodetectors, both in terms of high responsivity and rapid detection [23,24,25]. Graphene has already been produced on a large scale, and the growth process is more mature than that in other two-dimensional materials. Combined with a large body of research dedicated to THz detection, this demonstrates the feasibility of the use of graphene in this field.

In the past decade, several researchers have demonstrated the potential of graphene for use in THz detection. In 2013, Guider et al. used photoreduction measurements to prove that graphene is suitable for application to THz detection [26]. In 2017, Yang et al. designed a graphene field effect transistor on a plastic substrate, making THz detection more flexible [27]. Aziz et al. increased the gain of an antenna array by tuning it with graphene [28]. Detection efficiency and sensitivity can be further improved by using some novel graphene metamaterial structure or low-loss long-range spoof surface plasmons mode (LRSPP) models [29,30]. In 2021, M. Asgari et al. used single- and polycrystalline graphene, grown by CVD, to achieve the thermal detection of THz waves at room temperature [31]. In 2022, Chen et al. combined metamaterials and graphene to create a wavelength- and polarization-sensitive THz wave detector [32]. Fakharian also designed a multi-functional THz antenna in 2022 based on voltage. This device can regulate the parameters of the antenna through voltage changes [33]. Although these innovative antennas improve the performance of graphene detectors, they cannot fully meet the demands of high sensitivity and high integration at the same time. Therefore, it is crucial to develop a simple and highly integrated antenna structure.

Here, we design and optimize a grid antenna that can be prepared by a simple semiconductor process. The simulation results show that the proposed antenna structure has a strong coupling effect on incident THz radiation. By integrating an optimized metal-mesh antenna with high-quality monolayer graphene, we have achieved a THz detector with excellent light detection efficiency, high sensitivity, fast response time, and solid operation in self-powered mode. Because of the advantages of simple structure and easy integration, the detector provides the basis for the realization of array antenna with a high integration degree.

## 2. Materials and Methods

In the early stages, the mechanical exfoliation method was used to prepare graphene, which can obtain it with fewer defects and high quality. However, this method is inefficient and not conducive to large-scale preparation. In this work, the solution transfer method is used to transfer the graphene grown by chemical vapor deposition (CVD) from a copper substrate to a substrate. The specific method is as follows: the graphene grown on a copper substrate is coated with polymethyl methacrylate (PMMA) by spin coating, and then the copper substrate is etched as the base with ferric chloride solution. The etching time is about twenty-four hours, and the sample is cleaned with dilute hydrochloric acid. Then, the graphene covered with PMMA is transferred to the prepared Si/SiO_2_ substrate and dried in the shade. Finally, the PMMA on the graphene surface is removed with acetone solution and dried at 70 °C. The key to successful graphene transfer is to thoroughly remove the residual copper on the graphene surface and prevent the generation of bubbles between the graphene and the substrate (see Figure S1a in Supporting Information).

**Figure 1:**
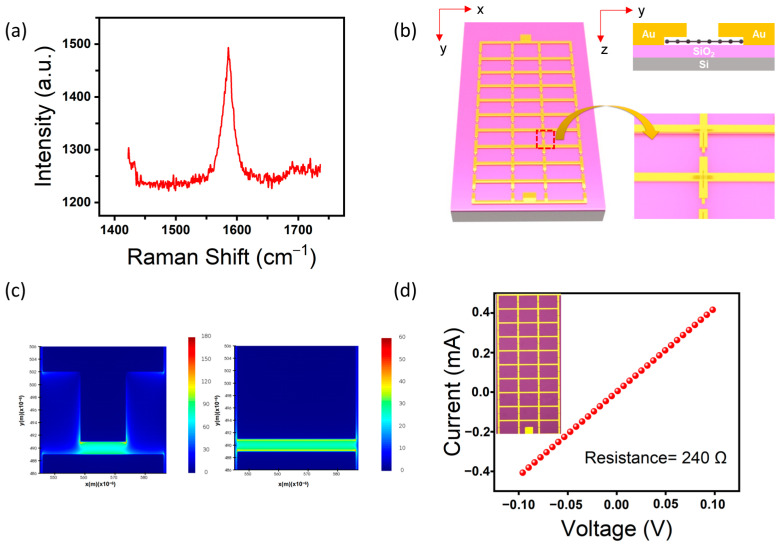
(**a**) The Raman spectroscopy characterization of graphene. (**b**) Schematic diagram of the structure for the grid antenna, and the illustration on the right is enlarged along the red dotted line. (**c**) By using FDTD simulation, the field distribution along channel of asymmetrical (left) and symmetrical (right) structures. (**d**) The current–voltage curve of the device is shown, and the insert presents the optical microphotography of the THz detector.

As shown in Figure 1a, Raman spectroscopy reveals that the graphene exhibits a peak at 1587 cm^−1^, in agreement with previous studies, attesting to its high quality and serving as a basis for the development of high-performance detectors [34,35]. Graphene height profile information is displayed in the Supporting Information (Figure S2). The structure of the detector is shown in Figure 1b, where the gridded antenna consists of multiple channels that are arranged in parallel along the x and y directions. The metal structure in contact with the channels and material is designed with an asymmetrical shape to increase the coupling efficiency of the antenna. For graphene-based THz detectors, where photothermal effects dominate, the sensitivity of the device can be improved by increasing the photovoltaic current under zero external bias voltage. The distribution of the THz light field in the gridded antenna is simulated and analyzed using finite-difference time-domain (FDTD) solutions, and the structure is further optimized to achieve strong coupling effects. The source shape is set as a plane wave, with polarization along the *y* axis. It is important that, for computational efficiency, the boundary conditions are set as periodic and the material comprising the antenna is assumed to be a perfect electrical conductor (PEC). The electromagnetic field distribution of the structure is shown in Figure 1c. The representation of the antenna in the terahertz domain is shown in Figure S3 of the Supporting Information. It can be observed that the electromagnetic field within the device channel region experiences a significant enhancement, which is accompanied by asymmetry. This indicates a desirable electromagnetic gain in the designed antenna structure. It is noteworthy that, aside from the asymmetrical design of the metal antenna structure, the graphene material is also etched into a congruent shape via oxygen plasma etching. The graphene-based detector we have proposed can be manufactured through a simple semiconductor process. Initially, the graphene film transferred onto a high-resistivity substrate is patterned into a specific shape using ultraviolet lithography, which is followed by oxygen plasma etching. The substrate chosen is highly resistive silicon with a 300 nm silica layer (resistance > 20,000 Ω·m), which avoids the degradation of device performance due to THz reflection from the substrate compared to a low-resistance substrate. After transferring the graphene, antennas are prepared by processes such as ultraviolet photolithography, electron beam evaporation, and lift-off processes (see Figure S1b in Supporting Information).

## 3. Results and Discussions

The electrical properties of the device were evaluated with a semiconductor parameter analyzer, as shown in Figure 1d. The current–voltage curve shows good linear behavior, which implies an ohmic contact between the graphene in the channel and the metal electrode. The device resistance is 240 Ω and the corresponding optical micrograph is shown in the inset. In order to further investigate the photoresponse characteristics of the detector at room temperature and the physical mechanism of its photocurrent generation, the optical properties of the device are characterized using a terahertz optical test platform. The terahertz radiation experiences fundamental frequency multiplication from the Agilent E8257D and is focused onto the detector through a set of lenses. The photocurrent is recorded by the computer after being passed through the pre-amplifier and the locked-phase amplifier chain.

**Figure 2:**
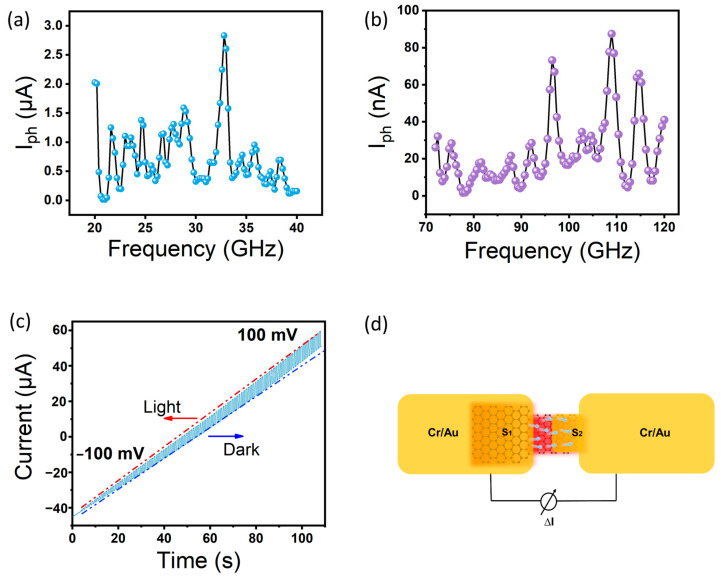
Photocurrent characteristics of the device. (**a**,**b**) show the frequency-dependent photoresponse of the device in the range of 0.02–0.04 THz and 0.08–0.12 THz. (**c**) Depicts the photoresponse change under different THz radiation states (light and dark). (**d**) Illustrates the schematic of the photothermoelectricity effect.

As shown in Figure 2a,b, multiple peaks in photocurrent occur in the frequency range from 0.02–0.04 THz to 0.07–0.12 THz at zero-bias voltage. The maximum photocurrent occurs at 0.032 THz and 0.11 THz, indicating a large gain effect of the graphene-integrated microstructure detector at a specific frequency. Figure 2c shows the variation in photocurrent as the bias voltage varies from −100 mV to 100 mV for radiation at 1 Hz modulation frequency during the dark state. A one-second period periodic sawtooth waveform appears when the detector is illuminated, and the device resistance changes significantly. Specifically, when the detector is unbiased, there is the generation of photocurrent. Conversely, combined with the high thermal conductivity properties of graphene, it is speculated that the main mechanism for photocurrent generation is the photothermoelectric (PTE) effect. The PTE effect is caused by the asymmetric radiation of incident light or the asymmetric antenna structure, which leads to a non-uniform distribution of temperature in the active region, resulting in a Seeback potential gradient, thus generating a photocurrent. To better explain the intrinsic physical mechanism, the principle diagram is represented in Figure 2d. The yellow area represents the metal antenna and the red area represents the temperature distribution in the active region after receiving radiation, which is mainly caused by the asymmetrical structure. The asymmetrical structure of the device results in a large optical response under zero-bias conditions. Incident terahertz waves are coupled through the antenna into the graphene channel, with strong terahertz wave absorption at the center of the channel, which gradually decreases on the central side, leading to the diffusion of hot carriers along the channel.

**Figure 3:**
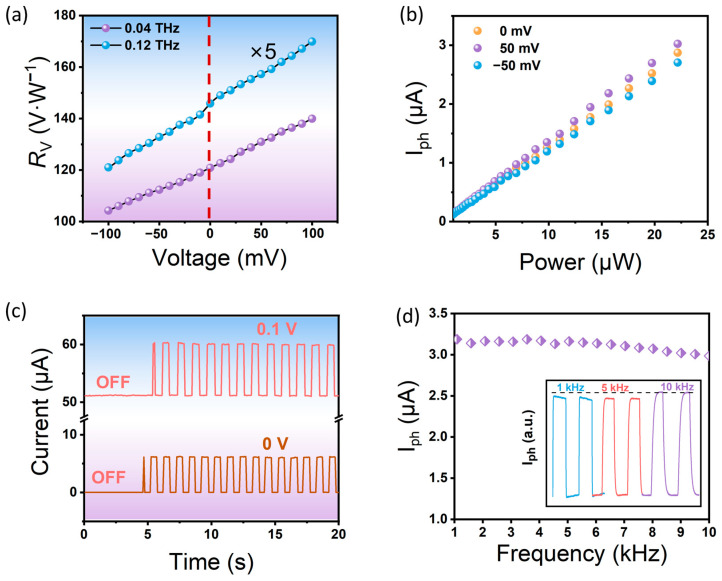
(**a**) The responsivity dependence on the bias of graphene-based THz detectors. (**b**) The photocurrent as a function of radiation power. (**c**) The time-resolved photocurrent of the detector under different biases. (**d**) The photoresponse dependence on modulation frequency, with the waveform of photoresponse shown for 1 kHz, 5 kHz, and 10 kHz.

In addition, the optical response of the device was studied at different power density and bias voltage conditions. In Figure 3a, the detector is applied with varying voltages which range from −100 mV to 100 mV and the optical response characteristics are measured at 0.04 THz and 0.12 THz of terahertz radiation, respectively. The results show that the microstructure-based tuned graphene terahertz detector can operate as a low-power detector and in self-powered mode. The photocurrent increases linearly with increasing bias voltage and does not reach saturation, which further supports the photothermal mechanism as the primary response mechanism. In Figure 3b, to verify the dynamic response range of the detector in the terahertz band, the variation in the photocurrent at different power densities is recorded. The graphene-based detector shows good linearity with increasing power density, and the photocurrent increases with the number of carriers in graphene. These results are consistent with previous reports on graphene-based terahertz detectors [31].

The asymmetric graphene effectively absorbed THz light under the modulation of grid-like microstructure, generating more photon-generated carriers. Then, the internal potential, driven by the temperature gradient, can effectively drive the nonequilibrium carrier, providing a basis for the device to generate high photocurrent values under zero-bias conditions. Our detector demonstrates high self-powered photo-detection ability under 0.04 THz and 0.12 THz radiation, offering a pre-condition for realizing arrayed monolayer graphene THz detectors. Responsivity is an important indicator for evaluating the sensitivity of photodetectors. Thus, the photocurrent voltage responsivity (*R_V_*) is calculated as:

(1) $$R_{V}=R_{A}\;\times \;r$$

(2) $$R_{A}=I_{\mathrm{ph}}/P_{0}\;\times \;S_{0}$$

where *R_A_* represents photocurrent responsivity, *r*, *I_ph_*, *P*_0_, and *S*_0_ represent resistance, photocurrent, power density, and effective area of the detector, respectively [36]. The responsivity at 0.04 THz and 0.12 THz are 120 V·W^−1^ and 29 V·W^−1^, respectively. Additionally, we validated that the device operates in self-powered mode, with good stability in the photocurrent waveform over time. Applying a small bias voltage of 0.1 V significantly improved the photocurrent, however, graphene’s inherent nature as a semi-metal inevitably increased the dark current, which is detrimental for detecting weak low-energy photons. Figure 3d also shows the time-resolved photocurrent curve of the device, indicating the excellent stability of the detector.

**Figure 4:**
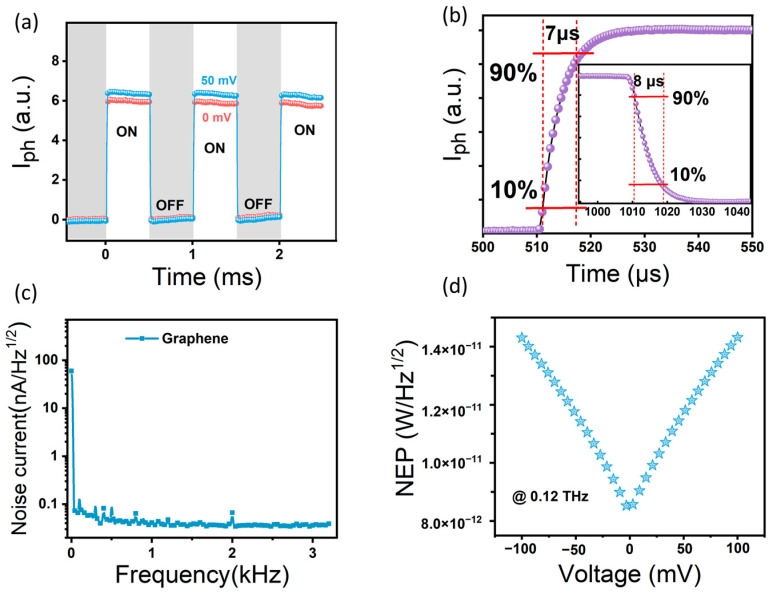
(**a**) Photoresponse waveform under different biasing conditions. (**b**) Response time of THz detector. (**c**) Voltage noise spectra of the device without biasing voltage. (**d**) Equivalent noise power of the device.

As shown in Figure 4a, the THz signal waveform collected by the detector using a high-speed oscilloscope at 0 mV and 50 mV demonstrates the obvious enhancement effect of the signal by applying a small bias voltage. It should be noted that the THz radiation output by the frequency-doubling circuit is modulated with a frequency of 1 kHz, and the modulation frequency is changed by internal electrical modulation in the microwave source, rather than by mechanical chopping. Compared to mechanical modulation, this method avoids the reflection of THz radiation caused by fan blade reflection and reduces the impact on the optical path. Response time is another key parameter of the photodetector, which reflects the detector’s ability to track rapidly changing light. Generally, the rise (or fall) time is defined as the time between 10% (or 90%) and 90% (or 10%) of the photocurrent to calculate. Figure 4b shows that the estimated rise and fall times are 7/8 μs, respectively. The fast response time demonstrated by this device results from the quick separation, transport, and effective recombination of carriers through the barrier. However, due to limitations in the bandwidth of the pre-amplifier and phase-locked amplifier, modulation constraints of the output source itself, and experimental error, the sub-microsecond response time does not fully represent the actual level of the graphene detector. Based on further optical characterization, the graphene photodetector based on microstructures exhibits a fast response time, good stability, and other characteristics. This is crucial for the future development of THz imaging and THz communication applications. The equivalent noise power (NEP) is also an important performance parameter for evaluating detector sensitivity, where $\mathrm{NEP}=\nu_{n}/R_{V}$, with $\nu_{n}$ being the noise spectral density and $R_{V}$ being the voltage responsivity. In the low frequency range, 1/f noise (flicker noise) dominates the noise current contribution (Figure 4c). In our test system, only the Johnson–Nyquist thermal noise (N_j_) and dark current-induced noise are considered [37,38], where $\nu_{n}$ is given by the expression:

(3) $$\nu_{n}={\left({\nu_{t}}^{2}+{\nu_{b}}^{2}\right)}^{1/2}={\left(4k_{B}T/r+2{\mathrm{qI}}_{\mathrm{dark}}\right)}^{1/2}$$

where *k_B_* is the Boltzmann constant, *T* is the thermodynamic temperature, *r* is the device resistance, *q* is the elementary charge, and *I_dark_* is the dark current. The smaller the value of NEP is, the better the performance of the detector will be. The NEP value calculated is only 8.5 pW/Hz^1/2^ without bias voltage (Figure 4d).

## 4. Conclusions

In summary, by introducing asymmetric optical coupling, we design microstructured antennas to achieve a non-uniform distribution of the terahertz local field. In addition, the artificial grid antenna can focus the terahertz field at the microscale, increasing the interaction between the monolayer graphene and terahertz light and achieving sensitive detection of low-energy photons. At room temperature, the graphene-based photodetector demonstrates a voltage responsivity of 29 V·W^−1^, fast response time (7 μs), and equivalent noise power of less than 8.5 pW/Hz^1/2^ in the self-powered mode. This work provides a viable approach to large-scale device integration and is one of the potential candidates for implementing graphene terahertz detection in focal plane arrays.

## Data Availability

Not applicable.

## Supplementary Materials

The following supporting information can be downloaded at: https://www.mdpi.com/article/10.3390/s23063203/s1, Figure S1: (a) Monolayer graphene transfer process. (b) Monolayer graphene terahertz detector preparation process; Figure S2: Height profile of the monolayer graphene tested by AFM; Figure S3: The antenna is characterised in the terahertz domain.
