# Supplementary material for: Monolayer Graphene Terahertz Detector Integrated with Artificial Microstructure
Source: Sensors (Basel). 2023 Mar 17;23(6):3203. doi: 10.3390/s23063203 (PMC10056542; doi:10.3390/s23063203)
Supplement: Supplementary file 1 [file sensors-23-03203-s001.zip › sensors-2271579-supplementary.pdf]

Supporting information

Monolayer Graphene Terahertz Detector Integrated with Artificial Microstructure

Mengjie Jiang [1,2], Kaixuan Zhang [1,2], Xuyang Lv [1,2], Lin Wang [2,*], Libo Zhang [2,3], Li Han[2,3], and Huaizhong Xing [1,*]

[1] Department of Optoelectronic Science and Engineering, State Key Laboratory for Modification of Chemical Fibers and Polymer Materials, DongHua University, Shanghai 201620, China

[2] State Key Laboratory for Infrared, Physics Shanghai Institute of Technical Physics, Chinese Academy of Sciences, 500 Yu-tian Road, Shanghai 200083, China

[3] College of Physics and Optoelectronic Engineering, Hangzhou Institute for Advanced Study, University of Chinese Academy of Sciences, No. 1, Sub-Lane Xiangshan, Xihu District, Hangzhou 310024, China

* Correspondence: wanglin@mail.sitp.ac.cn (L.W.); xinghz@dhu.edu.cn (H.X.)

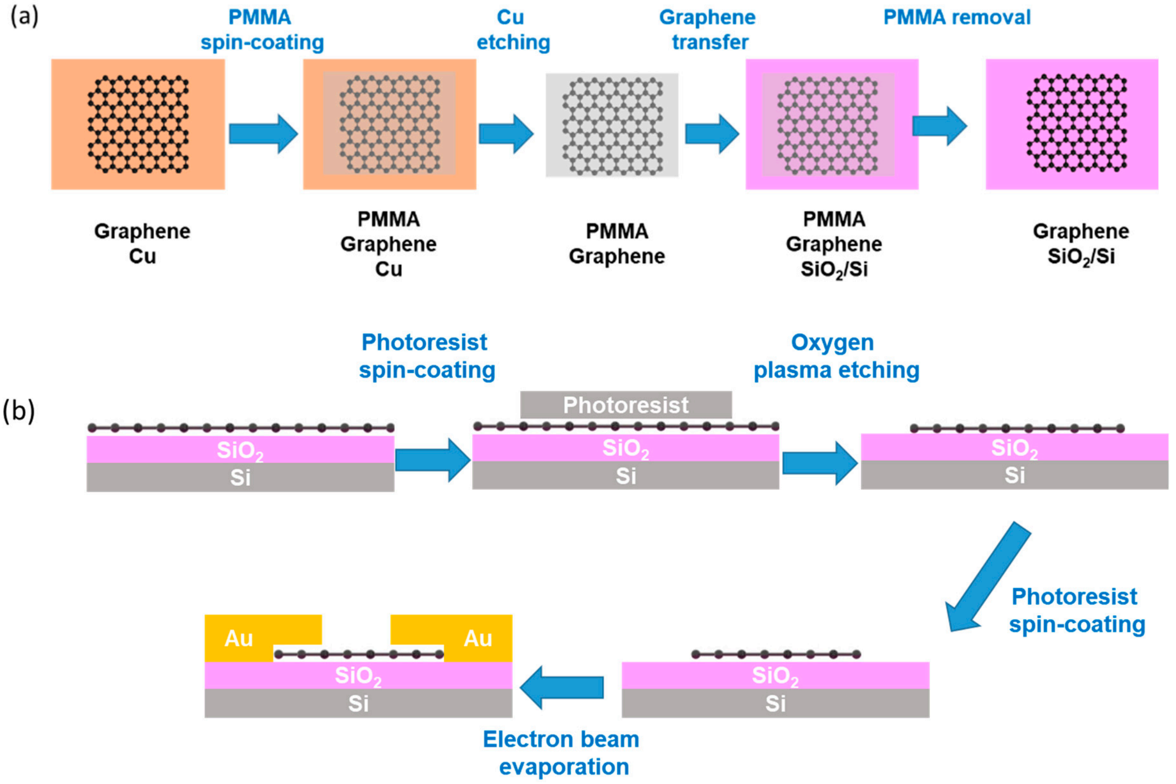

Figure S1. (a) Monolayer graphene transfer process (b) Monolayer graphene terahertz detector preparation process.

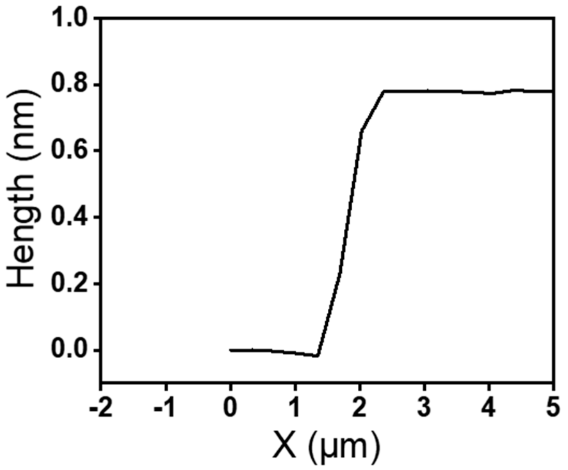

Figure S2. Height profile of the monolayer graphene tested by AFM.

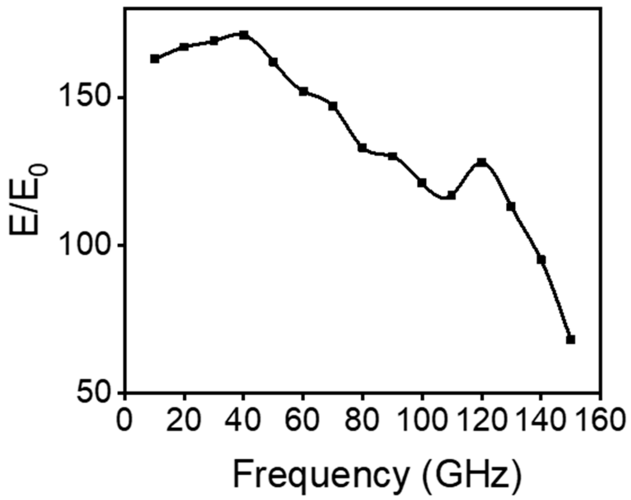

Figure S3. The antenna is characterised in the terahertz domain.
